# Examining disparities in the early adoption of Covid-19 personal mitigation across family structures

**DOI:** 10.3934/publichealth.2022041

**Published:** 2022-07-19

**Authors:** Casey T. Harris, Kevin Fitzpatrick, Michael Niño, Priya Thelapurath, Grant Drawve

**Affiliations:** 1 University of Arkansas, Department of Sociology and Criminology, 211 Old Main, Fayetteville, AR 72701; 2 Harvard University, Studies of Women, Gender, and Sexuality

**Keywords:** coronavirus, family, gender, risk, mitigation

## Abstract

The United States' response to the COVID-19 pandemic has relied heavily on personal mitigation behaviors versus centralized governmental prevention strategies, especially early in the virus's outbreak. This study examines how family structure shapes mitigation, focusing on the intersectional effects of gender, marital status, and the presence of children while accounting for differences in worry about infection from the virus. Using data from a national survey of 10,368 United States adults early in the pandemic (March 2020), survey-weighted logistic regression models show important differences in the likelihood of personal mitigation adoption across family structures. Unmarried women with children were most likely to report personal mitigation behaviors, including washing hands more frequently and avoiding social gatherings. Our findings highlight the differential impacts of the pandemic on those living in specific family circumstances.

## 1. Introduction

Since the first confirmed case in January 2020, the COVID-19 (coronavirus) pandemic has upended social, political, and economic life throughout the United States. As of early June 2022, over 84 million cases have been confirmed in the country, with more than 1 million recorded deaths [1]. The economic and social fallout of the pandemic has left millions unemployed, permanently closed many small businesses [2], and created significant uncertainty among the general population [3],[4]. Compared to nations that have relied upon centralized federal health policies (e.g., New Zealand, Germany, Norway), much of the United States' response has instead involved different state-level policies predicated on personal mitigation (e.g., washing one's hands more frequently, wearing a mask in public, avoiding social gatherings), particularly early in the pandemic [5],[6].

But who is more likely to adopt personal mitigation practices, such as handwashing and masking in public? Survey data reveal disparities across individuals' political identification, education, race, marital status, gender, the presence of children, and news consumption [7]–[11]. Yet, such snapshots mask important differences at the intersections of social statuses – for example, older men may adopt different risk-mitigation strategies from younger men or even women of their own age. Many of these descriptive polls also miss other important mechanisms that shape personal mitigation, such as worry about COVID infection or loss of income/employment. Such factors may make the likelihood of practicing such health behaviors more or less likely. The current study fills these gaps by examining how three dimensions of family structure – gender, marital status, and the presence of children – were associated with the adoption of coronavirus mitigation behaviors early in the pandemic.

We focus on family structure for three reasons. First, prior research reveals important disparities between men and women in health decision-making and risk mitigation, particularly in the context of marriage and raising children [12]. This literature finds that, outside of public health crises, family structure affects the way individuals manage health risk, and does so in ways that likely matter during the current COVID-19 pandemic. Second, much of the public discourse during the current pandemic focuses on particularly vulnerable individuals, including children whose risk is at least partially contingent upon family structure and family resources. That is, much of the media and public narrative of the pandemic has described risk in the context of individual and family responsibility [13],[14], but little is known about how family structure is associated with actual mitigation behaviors.

Third, emerging research on the social impacts of COVID-19 already finds family structure to be an important determinant of pandemic-related disparities in mental health and life satisfaction. For example, Calarco and colleagues [15] reveal pandemic-related increases in mothers' frustrations linked to dissatisfaction with partners' support and lack of concern from partners about infection. Similarly, other research shows that burden of care disparities between men and women have grown because of school closings and labor force contractions that have impacted mothers more than fathers [16],[17]. In particular, single mothers report greater stress associated with managing competing demands for their attention that have culminated in “pandemic exhaustion” [18]. Unlike many other localized catastrophes (e.g., natural disasters), the pandemic's social upheavals have fallen disproportionately on some individuals and their families in ways that need to be further investigated [19].

Our goal is to examine differences in early pandemic personal mitigation adoption across family structures defined by gender, marital status, and the presence of children. We begin by, first, reviewing existing literature on health decision-making and risk behaviors with a focus on gender, marital status, and the presence of children. Second, we articulate hypotheses for the adoption of COVID-19 mitigation followed by, third, a description of the current study. Fourth, we describe key findings from a unique survey of over ten-thousand United States adults conducted in March of 2020, before discussing the implications of these findings for public health outreach, advocacy, and for health inequality research.

### 1.1. Family structure and health decision making

A now sizeable literature emphasizes that risky behaviors – or, conversely, the mitigation of risk – vary across social positions, including those that define family structures (e.g., gender, marital status, and caring for children). Drawing on the social vulnerabilities framework of health [20], including research focusing on natural disasters [21],[22], this perspective emphasizes that individual demographic, social, and economic risks/resources are consequential for health outcomes. Such vulnerabilities affect both an individual's and community's capacity to respond to and lessen health risks, including the mitigation strategies that have been proposed during the COVID-19 pandemic.

We focus here on family structure as captured by gender, marital status, and the presence of children. First, gender remains a key stratifying dimension of health. For example, women are more likely than men to take on the responsibility of caring for elderly parents [23],[24]. Not surprisingly, women bear a disproportionate burden of care over their lifetimes as compared to their male counterparts [25]. Research indicates women are more likely to engage in certain health improvement activities like cancer screenings [26], utilize preventative health services [27], and worry about health risks generally, including during the COVID-19 pandemic [28]. More broadly, women are less likely to take risks and are more likely to mitigate them when possible [29].

Second, marital status also shapes population health and health-seeking behavior. Marriage has long been linked to better physical and mental health [30], even when compared to other family structures, such as cohabitation [31]. Yet, this relationship remains complex. On the one hand, those who marry may take their health more seriously and seek out preemptive care [30], gain access to insurance [32], or increase formal healthcare utilization [33]. On the other hand, those that are unmarried may adopt personal health improvement strategies that don't require formal healthcare resources, even if there is little long-term health gain [34],[35]. As it pertains to pandemic mitigation, this suggests that married individuals may have more structural resources that reduce their risk of illness, while those that are unmarried may use lower-cost personal mitigation strategies to reduce their risk in order to avoid incurring healthcare costs.

Third, the presence of a child has been shown to affect diet and exercise of parents and caregivers [36]. Likewise, becoming a parent, especially in conjunction with a partnership, leads to more general risk aversion [37]. More broadly, household health decisions with children present only affect the person making them, but others who may be at greater/lesser risk, as well [12]. In sum, the presence of children in a household may heighten perceptions of risk and encourage health mitigation behaviors among all household members.

### 1.2. Family structure and COVID-19 risk mitigation: hypotheses

Our review of health decision making and risk mitigation literatures suggests the following hypotheses as they pertain to early COVID-19 mitigation strategies:

H_1_: Women will report a greater likelihood of adopting personal coronavirus mitigation strategies than men.

H_2_: Those who are married will report a greater likelihood of adopting personal coronavirus mitigation strategies than those who are unmarried.

H_3_: Individuals with children in the household will report a greater likelihood of adopting personal coronavirus mitigation strategies than those without children present.

Additionally, we test two competing hypotheses, given how these statuses often overlap. On the one hand, the intersectional nature of gender, marital status, and the presence of children could be a driver of mitigation practices. Put simply, gender, marital status, and children could act as additive statuses that “stack” in ways that increase the likelihood of adopting COVID-19 mitigation.

H_4a_: Married women with children present will report a greater likelihood of adopting personal coronavirus mitigation strategies as compared to unmarried men without children.

On the other hand, the intersectional nature of these characteristics may work uniquely to affect the adoption of risk mitigation behaviors. For example, as noted above, unmarried adults may adopt personal health strategies that don't require formal healthcare resources, even if there is little long-term health gain [34],[35]. Simultaneously, child healthcare responsibilities (e.g., managing doctor's appointments or physically caring for them if they are sick) fall disproportionately to women [38]. This would suggest that unmarried women caring for children could be particularly likely to adopt personal mitigation strategies because of particular caregiving burdens faced by unmarried mothers especially [16],[17]. Likewise, as unmarried women with children – even compared to unmarried men with children – often possess fewer social support and resiliency resources [39], personal risk mitigation may be taken more seriously to avoid infection, labor force absence, missed schooling, and large medical bills. Scholarship showing that unmarried women experience higher levels of family stress generally [40] and during the pandemic specifically [18] supports this claim.

H_4b_: Unmarried women with children present will report a greater likelihood of adopting personal coronavirus mitigation strategies as compared to unmarried men without children.

## 2. Materials and methods

### 2.1. Data source

Data for the current study were drawn from a sample of adults (ages 18 and over) who completed an online, IRB-approved survey released on March 23, 2020, through Qualtrics Inc. to a national panel of United States residents (University of Arkansas human subjects approval #2003256438, 3/19/2020). The overall goal of this study was to explore the diffusion of fear and perceived threat across United States communities during the early pandemic period. Participants indicated consent for participation at the start of the survey with subsequent questions capturing general threat, fear, and anxiety related to COVID-19, physical and mental health assessments, and basic demographics. All final responses were required to have complete data (i.e., no missing values) and the final sample of 10,368 was completed on March 30, 2020. Post-stratification weights by gender, age, race, income, and geography (state) were applied to ensure the equitable contribution of respondents across their demographic and geographic strata relative to their representation in the overall population of the United States.

By the time the survey responses were fully collected on March 30, 2020, there were 161,575 confirmed cases in the United States, an increase of about 3.7 times as many cases as when the survey was released (n = 43,421) [41]. As such, these data captured individual behaviors at an early but strategic stage of the COVID-19 pandemic when there was little to no consistency in messaging about its threat but before widespread saturation of the virus. Importantly, these data were also able to account for early differences in perceived worry about the virus (net of marital status, gender, and the presence of children) that might impact whether some groups choose to adhere to personal mitigation recommendations in ways that affect infection risk.

### 2.2. Dependent variables

We examined four dependent variables reflecting common personal mitigation strategies emphasized during the earliest stages of the coronavirus pandemic. All respondents were asked to consider which steps they had taken “to prepare for the possibility of many cases of the coronavirus (COVID-19) in [their] community,” including whether they were (1) washing their hands more frequently, (2) avoiding social events/gatherings, (3) avoiding public transit, or (4) staying home from work. All four variables were dummy-coded (1 = respondent adopted a specific mitigation strategy; 0 = they did not adopt this strategy). Because these data were collected early in the COVID-19 outbreak, mask-wearing and vaccine uptake are not included as response options.

### 2.3. Family structure measures

To address disparities in the adoption of personal mitigation, we explored the intersections of three statuses that define family structure: gender, marital status, and the presence/absence of children in the household. Respondents were asked to separately indicate their gender (man, woman, and other), as well as whether they were married versus unmarried (combining divorced, separated, widowed, never married) and whether they currently had children under age 18 living in their household (yes, no). Using these variables, we constructed eight categories: (1) unmarried men with children, (2) unmarried women with children, (3) married men without children, (4) married women without children, (5) married men with children, (6) and married women with children, and (7) unmarried women without children. The eighth category, unmarried men without children living in the household, served as the reference (omitted category) for all multivariable analyses.

### 2.4. Additional control variables

In addition to the family structure statuses captured above, we controlled for age in years; income using dummy variables for less than $25k, $25k–$35k, $50k–$75k, $75k–$100k, $100k–$150k, and more than $150k; race/ethnicity dummy variables for non-Hispanic Black, Hispanic, non-Hispanic Asian, non-Hispanic Native American, and non-Hispanic other race (non-Hispanic White as the reference); and a dummy variable measuring whether a respondent is unemployed. Additionally, we also included a dummy measure of COVID-19 worry that reflects “how worried you are that you or your family will become infected with COVID-19” (all respondents indicating that they were “somewhat” “very” or “extremely” worried = 1, while “not all worried” or “a little worried” = 0). Finally, because surveys reveal disparities in worry about the virus and adoption of personal mitigation strategies across political identification (Pew Research Center 2020), we included dummy variables for Republican and Independent with Democrat serving as the reference.

### 2.5. Analytic method

Our analysis unfolded in two steps. First, we examined the overall distribution of our weighted sample of 10,368 respondents across our dependent and independent variables, noting the overall prevalence of early personal mitigation strategies. Second, we created a series of four survey-weighted logistic regressions models predicting whether respondents indicated they were washing their hands more frequently, avoiding social events, avoiding public transit, or staying home from work. We estimated all models in Stata 15 using the *svyset* and *svy* estimation procedures to accommodate the appropriate weights. Broadly, our goal in this second step of the analysis was to compare the likelihood of adopting each coronavirus mitigation strategy for each family structure classification, net of other important covariates.

## 3. Results

### 3.1. Descriptive statistics

Table 1 displays the descriptive statistics for our sample. All proportions, means, and standard deviations used survey weights to adjust each relative to their representation in the overall population of the United States as described above. We note three key findings.

First, the adoption of personal mitigation strategies was high even early in the pandemic. For example, over 80 percent of respondents reported avoiding social events and nearly 90 percent were washing their hands more frequently. Over 60 percent responded that they were avoiding public transit. The only mitigation strategy without majority adoption among respondents was staying home from work, though this could have reflected (a) that the survey was administered before many states had lockdown or stay at home orders in place or (b) that some respondents were essential workers and unable to adopt this approach.

Second, there were substantial differences in the prevalence of gender-by-family types. For example, men and women without children constituted the largest, but similarly sized, groups in our survey data at 22.2 and 22.8 percent, respectively. Likewise, married men and women without children represented similar 16.0 and 14.0 percent of respondents, just as married men with children (7.7 percent) and married women with children (7.6 percent) were comparable even if less prevalent. In contrast, unmarried women with children were twice as prevalent (6.6 percent) as unmarried men with children present (3.1 percent).

Third, our sample of respondents had a mean age of 47.44, was fairly evenly distributed across income categories, and was about 61 percent White, 18 percent Hispanic, and 12 percent Black or African American. For personal mitigation adoption, a large percentage of respondents (44.9 percent) were somewhat, very, or extremely worried (versus “not all all worried” or “a little worried” about COVID-19 during this mid-to-late March 2020 period. Finally, our sample was roughly evenly split among political ideologies, with a slightly higher percentage of Democrat respondents.

**Table 1.** Descriptive statistics for early COVID-19 pandemic sample (n = 10,368).

	Mean (SD)	Percentage
*Dependent Variables:*		
Washing Hands More Frequently	-	89.6%
Avoiding Social Events	-	80.5%
Avoiding Public Transit	-	62.1%
Staying Home from Work	-	45.6%
*Family Structure:*		
Unmarried Men, No Children	-	22.2%
Unmarried Men w/ Children	-	3.1%
Married Men, No Children	-	16.0%
Married Men w/ Children	-	7.7%
Unmarried Women, No Children	-	22.8%
Unmarried Women w/ Children	-	6.6%
Married Women, No Children	-	14.0%
Married Women w/ Children	-	7.6%
*Control Variables:*		
Age	47.44	-
Income: <$25k	-	23.9%
Income: $25–$35k	-	13.3%
Income: $35–$50k	-	13.4%
Income: $50–$75k	-	17.5%
Income: $75–$100k	-	12.9%
Income: $100–$150k	-	11.6%
Income: >$150k	-	7.4%
White	-	60.8%
Black	-	12.4%
Hispanic	-	18.2%
Asian	-	5.5%
Native American	-	0.6%
Other Race	-	2.5%
COVID-19 Worry	-	44.9%
Unemployed	-	19.6%
Democrat	-	34.7%
Republican	-	32.3%
Independent	-	33.0%

*Note: Income is displayed here in its respective categories but is treated as continuous in all subsequent models for the sake of parsimony. Models using six dummy categories and an omitted reference for income produce identical results.

### 3.2. Predicting personal mitigation adoption

**Table 2.** Logistic regression of personal mitigation on family structure and controls (n = 10,368).

	Washing Hands More Frequently		Avoiding Social Events	
	b	OR	b	OR
Unmarried Men w/ Children	0.20	1.22	0.36	1.43
	(0.37)			
Married Men, No Children	−0.03	0.97	0.11	1.11
	(0.16)		(0.13)	
Married Men w/ Children	0.14	1.15	0.01	1.00
	(0.21)		(0.14)	
Unmarried Women, No Children	0.30	1.34	0.49***	1.62
	(0.20)		(0.18)	
Unmarried Women w/ Children	0.78**	2.18	0.88***	2.42
	(0.53)		(0.49)	
Married Women, No Children	0.39	1.47	0.30*	1.35
	(0.30)		(0.19)	
Married Women w/ Children	0.15	1.15	0.32*	1.38
	(0.24)		(0.23)	
Age	0.02***	1.02	0.02***	1.02
	(0.01)		(0.01)	
Income	0.06	1.05	0.08***	1.08
	(0.03)		(0.02)	
Black	−0.46**	0.63	−0.47***	0.62
	(0.10)		(0.07)	
Hispanic	−0.30	0.74	−0.21	0.81
	(0.13)		(0.10)	
Asian	0.12	1.12	0.17	1.18
	(0.22)		(0.22)	
Native American	−0.72	0.48	−0.52	0.59
	(0.26)		(0.26)	
Other Race	−0.13	0.87	−0.48	0.61
	(0.38)		(0.17)	
COVID-19 Worry	0.73***	2.07	0.61***	1.84
	(0.22)		(0.14)	
Unemployed	−0.04	0.95	−0.05	0.94
	(0.12)		(0.10)	
Republican	−0.32*	0.72	−0.40***	0.67
	(0.10)		(0.06)	
Independent	−0.41***	0.66	−0.23*	0.79
	(0.08)		(0.07)	
Constant	0.90***		0.02	
	(0.59)		(0.18)	

*Note: Standard errors in parentheses. *p < 0.05, **p <0.01, ***p < 0.001. OR = odds ratios.

**Table 3.** Logistic regression of personal mitigation on family structure and controls (n = 10,368).

	Avoiding Public Transit		Staying Home from Work	
	b	OR	b	OR
Unmarried Men w/ Children	0.37	1.45	0.05	1.05
	(0.31)		(0.27)	
Married Men, No Children	0.02	1.02	−0.01	0.99
	(0.10)		(0.10)	
Married Men w/ Children	−0.01	0.99	0.26*	1.29
	(0.12)		(0.16)	
Unmarried Women, No Children	0.07	1.07	0.12	1.12
	(0.09)		(0.10)	
Unmarried Women w/ Children	0.468**	1.59	0.42**	1.52
	(0.24)		(0.21)	
Married Women, No Children	−0.08	0.91	0.05	1.04
	(0.09)		(0.10)	
Married Women w/ Children	−0.15	0.86	0.32**	1.37
	(0.11)		(0.16)	
Age	0.01***	1.00	−0.01***	0.98
	(0.01)		(0.01)	
Income	0.05**	1.05	0.17***	1.19
	(0.02)		(0.02)	
Black	−0.04	0.95	0.02	1.01
	(0.09)		(0.10)	
Hispanic	0.25*	1.28	−0.10	0.90
	(0.12)		(0.08)	
Asian	0.36**	1.43	0.66***	1.92
	(0.19)		(0.26)	
Native American	0.34	1.40	0.23	1.26
	(0.54)		(0.48)	
Other Race	0.06	1.06	0.47	1.60
	(0.27)		(0.41)	
COVID-19 Worry	0.61***	1.83	0.33***	1.39
	(0.11)		(0.08)	
Unemployed	−0.12	0.89	0.66***	1.92
	(0.07)		(0.17)	
Republican	−0.29***	0.75	−0.34***	0.70
	(0.05)		(.05)	
Independent	−0.18*	0.83	−0.24**	0.78
	(0.06)		(0.05)	
Constant	−0.32*		−0.47**	0.62
	(0.11)		(0.10)	

*Note: Standard errors in parentheses. *p < 0.05, **p < 0.01, ***p < 0.001. OR = odds ratios.

Tables 2 displays the results of the survey-weighted logistic regression models predicting whether a respondent reported washing their hands more frequently or avoiding social events. Table 3 includes the models predicting whether individuals indicated they were avoiding public transit or staying home from work. Unstandardized logit coefficients with their standard errors, as well as odds ratios (for ease of interpretation), are displayed for each of the four models. We note four key findings.

First, and central to our research question, there were statistically significant and substantively important disparities in the odds of personal mitigation adoption across respondents in different family structures. For washing one's hands more frequently, only unmarried women with children present were more likely to do so than unmarried men with no children. For avoiding social events or gatherings, all women respondents regardless of marital status and presence of children reported greater likelihoods (p < 0.05), though unmarried women with children were especially more likely than unmarried men without children to do so. In predicting whether respondents avoid public transportation or stay home from work, unmarried women with children were again more likely to do both than unmarried men without children, though married women with children and married men with children were also more likely than unmarried men without children to stay home from work.

**Figure 1.**
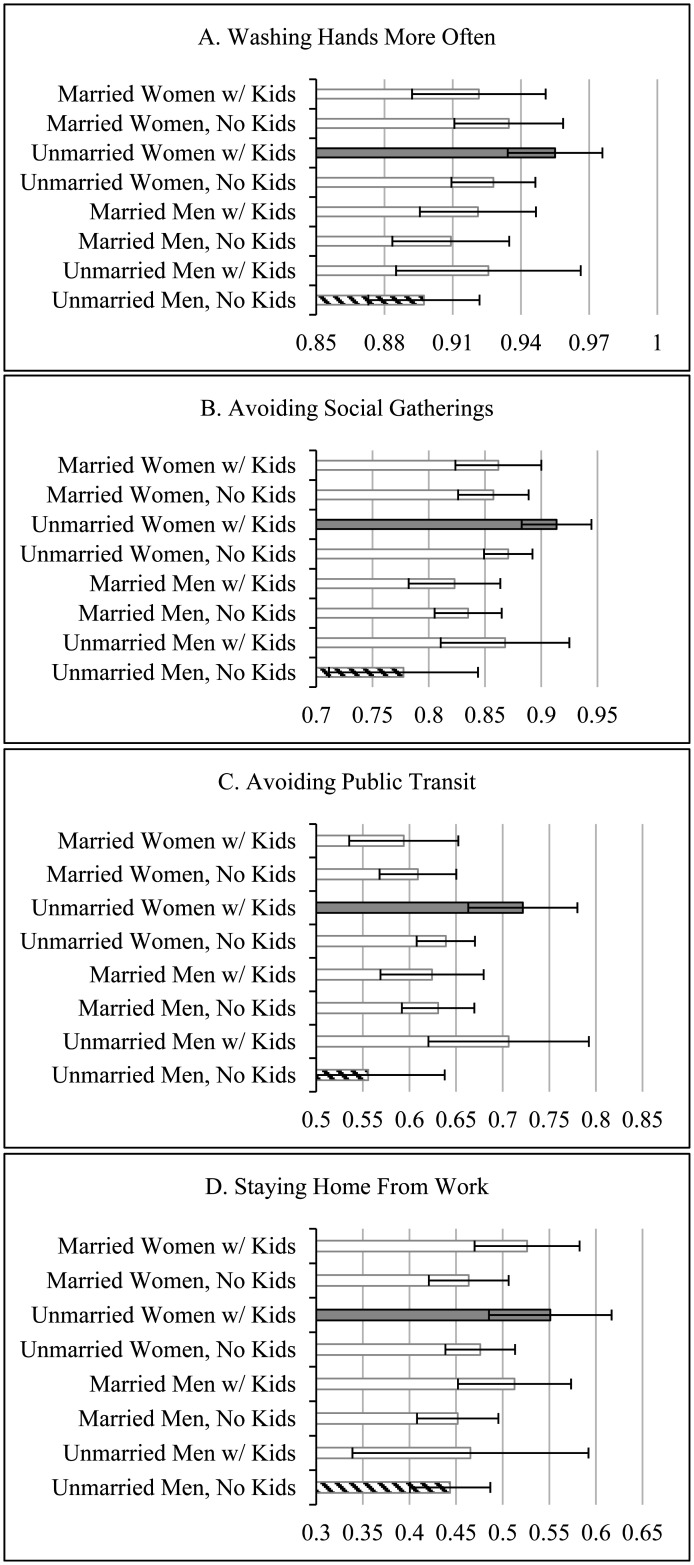
Predicted odds of mitigation strategies by family structure.

Most notably across these family structure configurations, unmarried women with children were most likely to adopt personal COVID-19 mitigation strategies early in the pandemic. Indeed, compared to unmarried men without children, they were the group that most consistently reported a greater likelihood of washing their hands frequently (p < 0.01), avoiding social events/gatherings (p < 0.001), avoiding public transit (p < 0.001), and staying home from work (p < 0.01), holding constant other important covariates. These were also sizeable disparities: the odds of unmarried women with children adopting such behaviors were between 50 and 142 percent greater than unmarried men with no children. Figure 1 helps to visually illustrate the greater likelihood, plotting the predicted odds for each family structure type, holding all other variables constant at their means. Note that the striped bars represent the reference group of unmarried men without children, while the gray shaded bars reflect unmarried women with children that emerged in Tables 2 and 3 as particularly likely to adopt mitigation behaviors.

A second finding is that there were disparities in the likelihood of adopting personal mitigation strategies across other demographic dimensions. Older respondents were more likely to wash their hands more frequently, avoid social events, and stay home from work (though there is a somewhat smaller likelihood they will avoid public transit), while those with higher incomes were more likely to adopt personal mitigation strategies except washing their hands more frequently. Holding constant other key covariates, there were some small but notable racial disparities: Black respondents were less likely to avoid social gatherings or wash their hands more frequently (p < 0.01), while Hispanics were slightly more likely to avoid public transit during this early pandemic period.

Third, worry or concern about the COVID-19 virus precipitated a greater likelihood of adopting all four personal mitigation outcomes. Those who reported that they are somewhat, very, or extremely worried that they or their family will become infected with COVID-19 are between 39 and 107 percent more likely to adopt each strategy (p < 0.001), with other factors statistically controlled. Such a finding illustrates the importance of tapping into underlying concerns in examining whether individuals personally work to offset risk (see also our robustness checks and supplemental models below).

Fourth, there were consistent and sizeable disparities in mitigation adoption by political ideology. Both self-identified Republicans and Independents were less likely to report washing their hands more frequently, avoiding social events or gatherings, avoiding public transit, or staying home from work (p < 0.05). Compared to Democrats, Independents had between 17 and 34 percent lower odds of adopting such behaviors, while Republicans had between 26 and 33 percent lower odds. Such disparities might have reflected differences in media consumption that emphasized personal mitigation efficacy more for Democrats than Republicans, where the latter may have been more likely to listen to or watch programming that downplayed the severity of COVID-19. Alternatively, such disparities might have reflected differences across geographic space in terms of virus saturation whereby many of the hardest-hit areas at this period of time included Democratic states (e.g., Washington, New York) where the early adoption of mitigation was more likely to be reported.

### 3.3. Robustness checks and supplemental models

To assess the robustness of our models to different specifications, we conducted supplemental analyses that (a) removed all unemployed individuals from analysis to avoid the potential for those respondents not working to skew the results for the model predicting the odds of staying home from work; (b) constrained the data to include only respondents in larger, metropolitan counties (i.e., percent urban greater than 70 percent); (c) estimated hierarchical logistic regression models that control for all variables currently estimated with the addition of spatial proximity of confirmed COVID-19 cases at the time of survey close (Queen's 1^st^ order, which captures the average Covid-19 case rate of all counties that touch each respondent's own county); (d) replaced the measure of COVID-19 worry with alternative measures of subjective fear (on a scale from zero to 10, how fearful are you of COVID-19); and (e) employed state-clustered standard errors to account for shared variance across geographic space. All models provided substantively similar results. Likewise, our other key patterns by age, income, race, and political ideology remained stable in these supplement models.

As a supplement to our primary analysis, we also examined whether specific family structures were more likely to report worry about COVID-19 infection and, in turn, whether worry might mediate the relationships between family structure and mitigation adoption. We found that, compared to unmarried men without children, married men with children were much more likely to report being worried, followed by married women with children, and married women without children. Unlike our mitigation adoption results shown in Tables 2 and 3, unmarried women with children were no more worried than unmarried men without children. Subsequently, examining models that excluded and then included worry about COVID-19 revealed no substantive differences in the family structure coefficients, indicating that adoption of personal mitigation strategies is not explained away by differences in worry about infection.

## 4. Discussion

The goal of the current study was to explore the ways that family structure shaped the adoption of personal mitigation strategies during the earliest phase of the COVID-19 pandemic. Because of how gender, marital status, and the presence of children impact health and healthcare decision-making broadly, we anticipated that these dimensions of family structure would similarly shape personal COVID-19 risk mitigation. Such expectations would complement emerging research showing disparities in a host of pandemic-related social and psychological outcomes across individuals' familial arrangements.

Broadly, we found that family structure played a role in COVID mitigation behaviors at the intersection of gender, marital status, and the presence of children. However, there was only limited evidence that women (per hypothesis 1) or individuals with children (per hypothesis 3) were more likely to adopt mitigation strategies. Likewise, we found limited support for the expectation that those who were married would do so (per hypothesis 2) and, critically, no support for hypothesis 4a that married women with children would be most likely to adopt mitigation behaviors. Instead, our strongest support was for hypothesis 4b: that unmarried women would be most likely to undertake personal risk mitigation strategies during the early stages of the COVID-19 pandemic. Such a finding was observed among respondent's, net of concern over the virus, socioeconomic resources, age, political identification, and other controls and for each of our four mitigation behaviors. Paralleling our findings, some prior research also finds that women are generally more likely to adopt “personal protection behaviours,” as are those who are married [42]. Yet, no study to-date examines the intersection of marital status, gender, and the presence of children simultaneously, so our finding that unmarried women with children are most likely to adopt mitigation behaviors remains unique.

Our findings could be explained by several factors. For example, those who care for children are more risk-averse generally [37] and to COVID-19 risks, particularly among female caregivers during the pandemic [28]. Simultaneously, unmarried women with children often possess fewer social support resources [39] which may be further exacerbated amidst job market contraction and an increased burden of care during the pandemic [16],[17]. Compared to other family circumstances, unmarried women with children may see personal COVID-19 mitigation as an easy way to avoid infection so as not to deplete the few resources at their disposal and further inflame “pandemic exhaustion” [18] by using healthcare services upon infection. With fewer social supports, unmarried women caring for children may see themselves as having few options but to take as many precautionary measures as they can.

Our findings also have important implications for public health outreach in suggesting that advocates and practitioners may find greater returns with targeted messaging directed toward those individuals and families least likely to have already adopted COVID-19 mitigation strategies (e.g., unmarried men without children). Clearly, some families have already adopted mitigation strategies, while others continue to lag behind in ways that provide ample room for improving the overall mitigation rate of the United States population. In turn, our findings suggest that different types of public health messaging may be required to encourage different types of families to take steps to mitigate COVID-19 risks. Likewise, our findings show that health inequalities research could benefit from a closer examination of family structure as it affects broader health patterns (versus examining gender, households with children, and marital status as discrete characteristics), including for current and future rates of COVID-19 vaccination.

Nevertheless, our study is not without limitations. Our data were collected early in the pandemic when the overall volume of cases and deaths was small, when vaccines were unavailable, as well as when the risks of the COVID-19 virus to specific groups (e.g., children) remained uncertain. While we see this as particularly valuable, additional research examining the intersections of gender, marital status, and the presence of children in later stages of the pandemic would be fruitful. For example, describing how individuals in unique family circumstances adopt personal mitigation or opt to receive vaccinations amidst face-to-face schooling would help public health practitioners better allocate resources designed to slow the spread of the virus at a time of changing public risk.

Additionally, the analysis presented here is largely descriptive and designed to examine whether there are differences in personal mitigation behavior across family structures. Extending this line of inquiry to explore why such differences emerge can be particularly useful for developing interventions that would engender greater public health engagement. For example, finding that some individuals adopt personal mitigation because they lack insurance and other formal healthcare resources or because they don't have family nearby to support them in the case of sickness would aid practitioners in bolstering mitigation messaging with other resources and services.

## 5. Conclusions

The COVID-19 pandemic continues to plague the United States even as vaccines are being distributed. As new mutations of the virus emerge (e.g., Delta, Omicron) and some vulnerable groups remain un- or under-vaccinated, the adoption of personal mitigation strategies will continue to be paramount. Likewise, mitigation behaviors like those studied here will remain important so long as vaccinated people remain vulnerable to ‘breakthrough’ infections. In many cases, decisions about whether to follow public health recommendations or not will be made by individuals whose unique family circumstances will shape that decision-making.
